# Alterations of serum macro-minerals and trace elements are associated with major depressive disorder: a case-control study

**DOI:** 10.1186/s12888-018-1685-z

**Published:** 2018-04-10

**Authors:** Md Rabiul Islam, Md Reazul Islam, M. M. A. Shalahuddin Qusar, Mohammad Safiqul Islam, Md Humayun Kabir, G. K. M. Mustafizur Rahman, Md Saiful Islam, Abul Hasnat

**Affiliations:** 1 Department of Clinical Pharmacy and Pharmacology, Faculty of Pharmacy, University of Dhaka, Dhaka, 1000 Bangladesh; 2 Department of Psychiatry, Bangabandhu Sheikh Mujib Medical University, Dhaka, 1000 Bangladesh; 3 Department of Pharmacy, Noakhali Science and Technology University, Sonapur, Noakhali, 3814 Bangladesh; 4 Department of Soil Science, Faculty of Agriculture, Bangabandhu Sheikh Mujibur Rahman Agricultural University, Salna, Gazipur, 1706 Bangladesh

**Keywords:** Major depressive disorder, Macro-minerals, Trace elements, Case-control, Inter-element relations

## Abstract

**Background:**

Major depressive disorder (MDD) is a mixed disorder with the highly irregular course, inconsistent response to treatment and has no well-known mechanism for the pathophysiology. Major causes of depression are genetic, neurobiological, and environmental. However, over the past few years, altered serum levels of macro-minerals (MM) and trace elements (TE) have been recognized as major causative factors to the pathogenesis of many mental disorders. The purpose of this study was to determine the serum levels of MM (calcium and magnesium) and TE (copper, iron, manganese, selenium, and zinc) in MDD patients and find out their associations with depression risk.

**Methods:**

This prospective case-control study recruited 247 patients and 248 healthy volunteers matched by age and sex. The serum levels of MM and TE were analyzed by atomic absorption spectroscopy (AAS). Statistical analysis was performed with independent sample t-tests and Pearson’s correlation test.

**Results:**

We found significantly decreased concentrations of calcium and magnesium, iron, manganese, selenium, and zinc in MDD patients compared with control subjects (*p* < 0.05). But the concentration of copper was significantly increased in the patients than control subjects (*p* < 0.05). Data obtained from different inter-element relations in MDD patients and control subjects strongly suggest that there is a disturbance in the element homeostasis.

**Conclusion:**

Our study suggests that altered serum concentrations of MM and TE are major contributing factors for the pathogenesis of MDD. Alterations of these elements in serum levels of MDD patients arise independently and they may provide a prognostic tool for the assessment of depression risk.

## Background

Major depressive disorder (MDD) is accompanied by low self-esteem and loss of interest or pleasure in day to day activities that adversely affect a person’s professional and personal life [1]. As major depression gives the enormous burden on an individual’s life, extensive efforts have been made to explore the biological mechanisms of it [2]. Major depression has been considered as a multifactorial disorder with genetic, neurological, and environmental factors contributing to overall risk. However, the mechanisms of these risk factors are still unknown [3]. Macro-minerals (MM) and trace elements (TE) play a versatile role in the biological system ranging from regulating metabolic reactions to acting as antioxidants [4, 5]. Several studies have suggested that alterations of these elements in serum levels are linked with the etiology and pathophysiology of many mental disorders [4, 6–9], including major depression [10].

Calcium (Ca) is essential for activation of different enzymes and plays a vital role in neuromuscular excitability. Low Ca level in the blood serum causes fragility of hair and nails and can also lead to mood disorders and depression [11]. Thus the deficiency of this mineral in the human body is linked with several chronic diseases [12]. Magnesium (Mg) acts as a coenzyme for many enzymatic systems. Mg is considered as an important factor in the treatment of depression due to its regulatory effects on N-methyl-D-aspartate (NMDA) channels [11]. Chronic stress, alcohol abuse, a diet rich in carbohydrates and fats cause Mg deficiency in the human and prolonged deficiency of this mineral develops depression [13].

Copper (Cu) accumulates in the liver, muscles, skeletal system, and brain of the human. Our kidneys, liver, and coronary arteries can be damaged by the high concentration of Cu in blood serum. Although, many disorders occur in the circulatory system, nervous system, and digestive system due to the deficiency of Cu [14]. In the human body, iron (Fe) transports oxygen through hemoglobin [15]. Fe deficiency is manifested mainly in weakness due to the limiting of aerobic changes in muscles. Moreover, some authors associate a decrease in body temperature, low appetite, and restless leg syndrome with Fe deficiency [16, 17]. Manganese (Mn) is a crucial trace element for human health [18]. In the central nervous system (CNS), Mn is presents in several proteins and key enzymes which are associated with some neurodegenerative disorders [19–21]. Selenium (Se) is an essential nutrient required for the functioning of antioxidant defenses in the brain and nervous system [22]. Zinc (Zn) is considered as an important element in the human body and sufficient amount of Zn is required for nucleic acid and protein metabolism, cellular growth, division and functions [23]. Zn is an important modulator of the functioning of the central nervous system [24].

Moreover, major depression can be influenced by some specific metalloenzymatic reactions in the brain. Trace metals regulate a range of cellular metabolic reactions and some of them are responsible for the etiology of several neurological disorders [25]. Superoxide dismutase (SOD) is a metalloenzyme that contains metal ions in its structure [26]. In human, three types of SOD are present. SOD_1_ is cytoplasmic, SOD_2_ is mitochondrial and SOD_3_ is extracellular. SOD_1_ is a dimer while SOD_2_ and SOD_3_ are tetramers. SOD_1_ and SOD_3_ contain Cu and Zn, while SOD_2_ contain Mn at their reactive center [27]. CuZnSOD involved in defense against reactive oxygen species (ROS). MnSOD is an antioxidant enzyme that provides protection against free radicals [28].

Based on the above observations, the present study was undertaken to explore the associations of serum MM and TE levels with the risk of major depression on Bangladeshi population.

## Methods

### Study design and blood sample collection

This prospective case-control study enrolled 247 MDD patients and 248 healthy individuals. The patients were recruited from the department of psychiatry, Bangabandhu Sheikh Mujib Medical University (BSMMU), Dhaka, Bangladesh but the controls were from different parts of Dhaka city matched by age, gender and body mass index (BMI) with the patients. A specialized psychiatrist diagnosed the cases and evaluated the controls according to the diagnostic and statistical manual of mental disorders, 5th edition (DSM-V). Detailed physical and neurological screenings were performed to diagnose the coexistence of other complications. The study subjects had no previous history of liver or kidney failure and had not been treated with any medication that could interfere with the concentrations of MM or TE. Patients with mental retardation and comorbid psychiatric illness were also excluded from this study. Additional exclusion criteria were alcohol and substance abuse or dependency, tardive dyskinesia related to neuroleptics, severe organic conditions, excessive obesity and presence of infectious diseases. Sociodemographic data were recorded by using pre-designed questionnaires. Different biographical features (height, weight) and BMI were also examined for both the cases and controls.

Blood samples (5 ml) were collected from the cephalic vein of each participant after an overnight fast. The samples were allowed to clot for one hour at room temperature. After centrifugation at 3000 rpm for 15 min, serum samples were extracted from the collected blood samples, placed into microtubes and stored at − 80 °C until analysis.

### Chemical and reagents

Analytical grade reagents were used for the study from the commercially available company. Standards of Ca, Mg, Cu, Fe, Mn, Se, and Zn were sourced from ABCR GmbH & Co. KG, Germany. Hydrochloric acid (37%) and nitric acid were purchased from Merck, Germany. Other supportive chemicals of recommended grade were supplied by clinical pharmacy and pharmacology department, University of Dhaka, Bangladesh.

### Determination of macro-minerals and trace elements

Serum level of MM and TE were measured by both flame atomic absorption spectrometry (FAAS) and graphite furnace atomic absorption spectrometry (GFAAS) following the method described in our previous articles [29, 30]. Briefly, collected serum samples were diluted with deionized water 1:10 dilution. Different concentrations of minerals (0.5, 1.0, 2.0, 5.0 and 10.0 mg/L) were used to prepare the calibration curve. Finally, the concentrations of MM and TE were measured by reading the absorbances’ at 422.7, 285.2, 327.4, 248.3, 279.8, 196.0 and 213.9 nm for Ca, Mg, Cu, Fe, Mn, Se, and Zn, respectively. The standard solutions were run for every 10 test samples to confirm the test precision and quality. The limits of detection (LoDs) were established by analyzing five blank solutions. The σ value was estimated by Microsoft office excel 2010 program. LoDs were found as follows (μg/L): ^40^Ca-1.9, ^24^Mg-0.24, ^63^Cu-1.8, ^56^Fe-0.13, ^55^Mn-0.07, ^77^Se-0.04, and ^66^Zn-0.05. SpectrAA software package was used to calculate the concentrations of MM and TE in serum samples using calibration curve. The safety measures for both collection and subsequent management of serum samples were taken to avoid or decrease MM and TE contamination.

### Statistical analysis

Serum levels of MM and TE were presented as the mean ± standard error mean (mean ± SEM) and compared between the cases and the controls with independent sample t-tests. Boxplot graphs were used to compare study parameters between the patient group and the control subjects. Correlations were established among different study parameters using Pearson’s correlation test. *p* < 0.05 was considered to be statistically significant. Statistical analysis was performed using SPSS statistical software, version 20.0 (Armonk, NY: IBM Corp.)

## Results

### Anthropometric and demographic profile of the study population

The study population was categorized based on their socioeconomic conditions, biophysical characteristics and smoking habit. Socioeconomic data of MDD patients and control subjects have been shown in Table 1 where female comprised the highest percentage of both MDD patients and control subjects than the male. It was found that most of the patients were literate (87%) and nonsmoker (73%). BMI values were normal for 84% patients and 78% control subjects. Among all MDD patients, 38% were very poor and 79% had average monthly family income ≤25 k Bangladeshi taka (KBDT). Only 7% patients had monthly family income above 40 KBDT. Statistical analysis showed that the differences of age, education, occupation, BMI, income and smoking habit were not significant between the groups (*p* > 0.05).

**Table 1:** Anthropometric and demographic profile of the study population

Parameters	Patients (*n* = 247)			Controls (*n* = 248)			*p* value
	*n*	%	Mean ± SEM	*n*	%	Mean ± SEM	
Age in years							
18–24	58	23		54	22		0.576
25–34	78	32	33.03 ± 0.693	79	32	33.55 ± 0.608	
35–44	65	26		74	30		
45–60	46	19		41	17		
Gender							
Female	156	63		147	59		0.193
Male	91	37		101	41		
BMI (kg/m^2^)							
Below 18.5 (CED)	23	9		25	10		0.193
18.5–25 (normal)	208	84	22.82 ± 0.161	194	78	23.15 ± 0.191	
Above 25 (obese)	16	6		29	12		
Education							
Illiterate	32	13		26	10		0.958
Can read only	47	19		51	21		
Secondary	31	13		35	14		
Higher secondary	63	26		67	27		
Graduate and above	74	30		69	28		
Occupation							
Service	22	9		21	8		0.673
Business	31	13		29	12		
Student	55	22		69	28		
Others	103	42		97	39		
Jobless	36	15		32	13		
Monthly income in KBDT							
Below 10	94	38		59	24		0.413
10–25	101	41	19.28 ± 0.899	97	39	20.33 ± 0.911	
26–40	34	14		75	30		
Above 40	18	7		17	7		
Smoking habit							
Nonsmoker	180	73		190	77		0.352
Smoker	67	27		58	23		

*CED* chronic energy deficiency, *KBDT* kilo Bangladeshi taka. **p < 0.05 (Significant difference between patient and control groups at 95% confidence interval)

### Serum levels of macro-minerals and trace elements

**Fig. 1:**
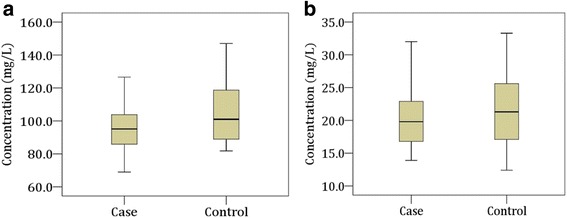
Changes in serum levels of macro-minerals in the study population. Boxplot showing the median, maximum and minimum value range. **a** Calcium, **b**: Magnesium

**Fig. 2:**
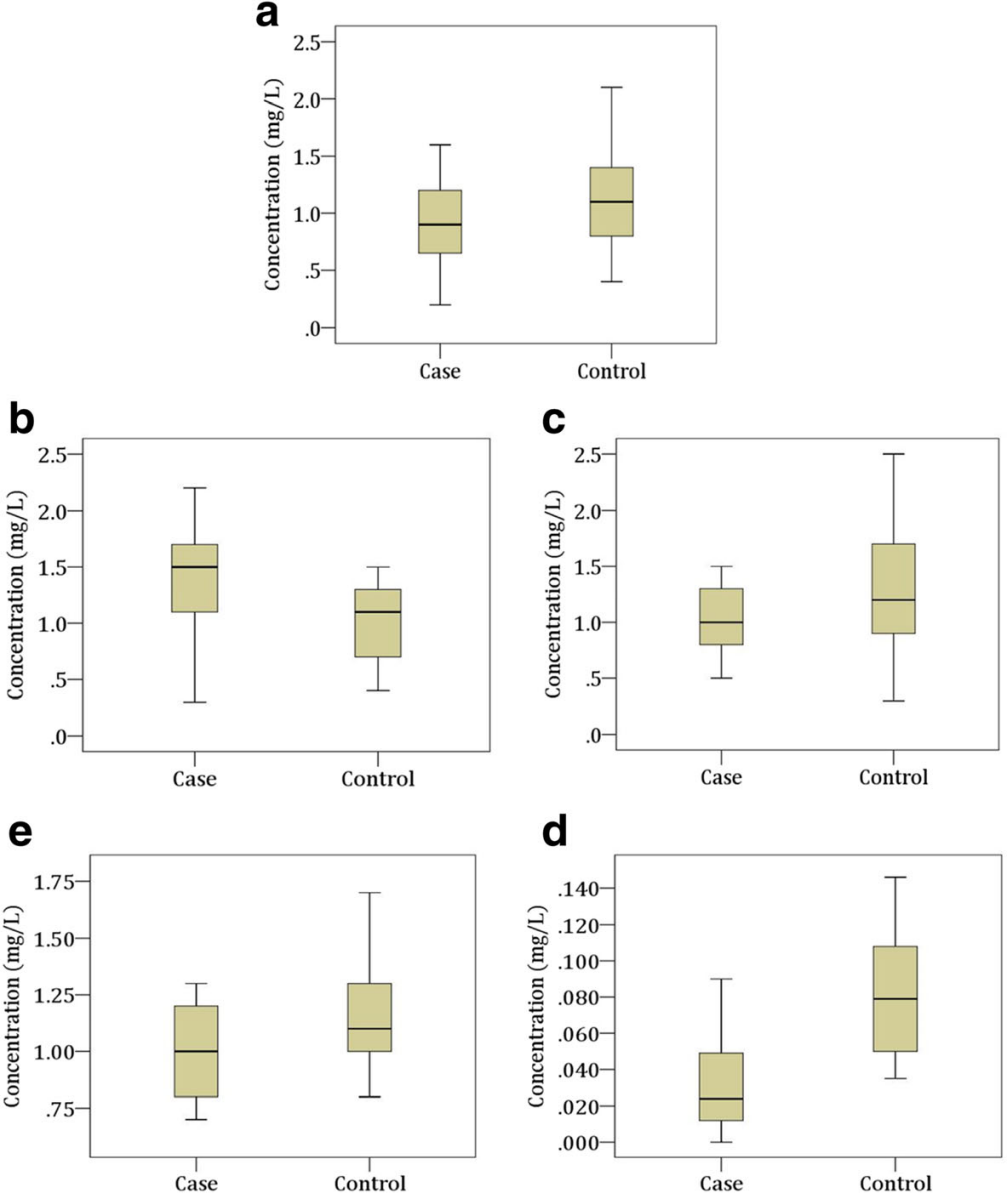
Changes in serum levels of trace elements in the study population. Boxplot showing the median, maximum and minimum value range. **a** Zinc, **b** Copper, **c** Iron, **d** Manganese, **e** Selenium

The mean serum concentrations of MM and TE for study population were presented in Table 2. Serum levels of Ca, Mg, Fe, Mn, Se, and Zn were found significantly decreased in MDD patients than control subjects (*p* < 0.05). But the concentration of Cu was significantly higher in the patient group (*p* > 0.05). The changes of serum MM and TE levels for cases and controls were presented in Figs. 1 and 2.

**Table 2:** Serum level of macro-minerals and trace elements in the study population

Elements (mg/L)	Values (Mean ± SEM)			
	Patient group	Control group	Reference range^a^	*p* value*
Ca	94.91 ± 0.85	105.06 ± 1.05	90–110	*p* < 0.05
Mg	20.37 ± 0.28	21.85 ± 0.34	18–36	*p* < 0.05
Cu	1.39 ± 0.03	1.01 ± 0.02	0.6–1.4	*p* < 0.05
Fe	1.02 ± 0.02	1.30 ± 0.03	1.1–1.3	*p* < 0.05
Mn	1.00 ± 0. 01	1.17 ± 0.01	0.1–2.9	*p* < 0.05
Se	0.03 ± 0.002	0.07 ± 0.003	0.06–0.11	*p* < 0.05
Zn	0.92 ± 0.02	1.09 ± 0.02	0.6–1.2	*p* < 0.05

*p < 0.05 (Significant difference between patient and control groups at 95% confidence interval)

^a^ Reference values are given in milligrams per liter unit from the handbook on metals in clinical and analytical chemistry [53, 54]

### Correlation study

Pearson’s correlation was used to establish inter-element relationships between the patients and controls for the investigated elements and presented in Table 3. Among all established relationships, patient group showed significantly negative correlations between Mn and Zn (*r* = − 0.184, *p* = 0.004), Mn and Cu (*r* = − 0.171, *p* = 0.007), Cu and Se (*r* = − 0.175, *p* = 0.006), statistically significant positive correlations were also observed between Mn and Mg (*r* = 0.155, *p* = 0.015), Mg and Se (*r* = 0.145, *p* = 0.023). Control group showed statistically significant (*p* < 0.05) negative correlations between Mn and Cu (*r* = − 0.275, *p* < 0.001), Cu and Mg (*r* = − 0.240, *p* < 0.001). All other positive and negative correlations between MM and TE were not found statistically significant for both of the study groups. Study parameters were not found significantly correlated with age and BMI of the study population.

**Table 3:** Correlation study among various research parameters in patient and control groups

Correlation parameters	Patient group		Control group	
	*r*	*p*	*r*	*p*
Fe and Mn	−0.031	0.630	−0.021	0.743
Fe and Zn	0.025	0.698	0.003	0.963
Fe and Cu	0.045	0.479	< 0.001	0.999
Fe and Ca	−0.070	0.272	0.006	0.919
Fe and Mg	−0.032	0.619	0.049	0.438
Fe and Se	−0.121	0.058	0.017	0.788
Mn and Zn	−0.184	0.004^a^	0.086	0.178
Mn and Cu	−0.171	0.007^a^	−0.275	< 0.001^a^
Mn and Ca	0.041	0.518	−0.030	0.641
Mn and Mg	0.155	0.015	0.266	< 0.001^a^
Mn and Se	0.059	0.357	−0.006	0.927
Zn and Cu	0.079	0.219	< 0.001	0.995
Zn and Ca	−0.035	0.589	0.072	0.260
Zn and Mg	0.032	0.613	−0.042	0.506
Zn and Se	−0.012	0.852	0.047	0.458
Cu and Ca	−0.043	0.505	0.093	0.145
Cu and Mg	0.078	0.220	−0.240	< 0.001^a^
Cu and Se	−0.175	0.006^a^	−0.008	0.905
Ca and Mg	−0.018	0.774	−0.004	0.952
Ca and Se	−0.004	0.944	−0.031	0.623
Mg and Se	0.145	0.023	−0.051	0.424

*r* = Correlation co-efficient; *p* = Significance; Negative values specify opposite correlation

^a^ Correlation is significant at 0.05 level (two-tailed)

## Discussion

To the best of our knowledge, this is the first ever study on Bangladeshi patients to find out the association of serum MM and TE levels with MDD. The required amounts of MM and TE are essential for maintaining proper healthy life. The present study explored the associations of MM and TE with the risk of major depression, demonstrating that alterations of serum MM and TE levels are associated with the increased risk of MDD. This generally happens when usual neurological physiology is troubled [31].

Several studies have shown that lower levels of serum Ca and Mg can cause various symptoms e.g. depression, anxiety, behavior, and personality changes [32]. One study showed that the significant deficiency of Ca was found in depression [33]. Other studies reported that serum concentrations of Mg were found substantially reduced in depressed patients [34, 35]. Another study revealed that Mg rich diet reduces the depressive symptoms [36]. The severity of depression is significantly influenced by the serum levels of Mg which confirms the involvement of Mg in the pathogenesis of depression [37]. In our current study, we found significantly lower levels of serum Ca and Mg in MDD patients compared to control subjects (*p* < 0.05). Thus this downregulation of serum MM levels may be involved in the pathogenesis of MDD.

The average concentration of serum Cu was found higher in depressed patients by 21% than healthy controls [38]. In our study, we also found the higher concentration of Cu in MDD patients compared with the control subjects (*p* < 0.05). Fe plays a major role in the development of the central nervous system (CNS). Moreover, it plays a crucial role in the development of depression as fatigue is caused by Fe deficiency. The current study found that the serum levels of Fe were reduced significantly in MDD patients which are supported by previous study results [39]. Low levels of Mn cause depression by increasing auto-immune reactions and macrocytosis [40]. In our present study, we found the significantly lower concentration of Mn in MDD patients compared with the control subjects (*p* < 0.05). Another valuable element is Se and any deficiency of this element causes the glutathione peroxidase dysfunction which is an enzyme that protects oxidative damage [41]. Also, Se modulates the status of many neurotransmitters [42]. Lower Se concentration is a risk factor for depression via antioxidant pathways [43]. According to our study, Se concentrations were significantly lower in MDD patients compared with the controls (*p* < 0.05). This evidence suggests that Se deficiency contributes to the pathogenesis of MDD as it prevents oxidative damage. Zn deficiency in humans is relatively rare but it occurs during the emotional stress and some disease conditions e.g. giardiasis, diarrhea, acute pancreatitis, and chronic renal failure. Several studies have suggested that prolonged Zn deficiency causes neuropsychiatric disorders such as depression and lack of concentration [43–45]. These observations are consistent with our present study result where a significantly decreased level of serum Zn was found in MDD patients (*p* < 0.05).

Nearly 7.6% patients suffering from psychiatric disorder have nutritional problems [46]. Serum concentrations of MM and TE are known to be influenced by dietary factors. The effects of diet on serum level of MM such as Ca and Mg are sparse [47]. Serum levels of Fe and Zn are especially affected by diet [48]. Serum level of Se could be affected by the low Se containing foodstuffs [49]. Deficiencies of Cu and Mn in the serum level are unusual due to a wide variety of dietary sources [50]. Pharmacotherapy is also a considerable factor that influences the serum levels of MM and TE in MDD patients. Serum Cu level is influenced by acute antidepressant therapy such as escitalopram and reboxetine reduced and imipramine increased serum Cu level [51]. Decreased serum Zn concentration can be normalized after successful antidepressant therapy e.g. citalopram increases serum Zn level [52].

At the end of our discussion, we can give some outline about lifestyle and diet as interventional events for MDD. According to our study findings, we can propose diet, current pharmacotherapy, and lifestyle as considerable factors for the proper treatment of MDD. We did not investigate the impact of dietary supplementation, current pharmacotherapy, and lifestyle on our study parameters that were the main drawback of our study. Therefore, this is a preliminary study and further investigation with more homogenous samples is required to support our findings. In spite of these limitations, the present study has some significant advantages. The first strength is the large study population match on age, sex and residential areas of patients and healthy controls. Another one is the simultaneous determination of all macro and micro elements under the same experimental conditions.

## Conclusion

The present study illustrates that MDD patients have reduced serum concentrations of MM and TE except for Cu compared with healthy volunteers. So these findings suggest the possible involvement of depleted serum MM and TE in the pathogenesis of depression. It was found that there was no significant correlation of serum MM and TE with age and BMI of the patient group. The reduced serum MM and TE may have an influence on the development of MDD. We thus recommend the altered serum levels of MM and TE are associated with the risk of MDD which may require further study.

## Abbreviations

AAS: Atomic absorption spectroscopy
AD: Alzheimer’s disease
BD: Bipolar disorder
BMI: Body mass index
BSMMU: Bangabandhu Sheikh Mujib Medical University
BSMRAU: Bangabandhu Sheikh Mujibur Rahman Agricultural University
CED: Chronic energy deficiency
CNS: Central nervous system
DSM-V: Diagnostic and statistical manual of mental disorders, 5th edition
GAD: Generalized anxiety disorder
KBDT: Kilo Bangladeshi taka
LoD: Limit of detection
MDD: Major depressive disorder
MM: Macro-minerals
OCD: Obsessive-compulsive disorder
PAM: Peptidylglycine α-amidating mono-oxygenase
ROS: Reactive oxygen species
SEM: Standard error mean
SOD: Superoxide dismutase
SPSS: Statistical package for the social sciences
TE: Trace elements

## References

[1] Wakefield JC, Schmitz MF, First MB, Horwitz AV (2007). Extending the bereavement exclusion for major depression to other losses: evidence from the National Comorbidity Survey. Arch Gen Psychiatry.

[2] Hsu KJ, Young-Wolff KC, Kendler KS, Halberstadt LJ, Prescott CA (2014). Neuropsychological deficits in major depression reflect genetic/familial risk more than clinical history: a monozygotic discordant twin-pair study. Psychiatry Res.

[3] Wilson S, Vaidyanathan U, Miller MB, McGue M, Iacono WG (2014). Premorbid risk factors for major depressive disorder: are they associated with early onset and recurrent course?. Dev Psychopathol.

[4] Alimonti A, Ristori G, Giubilei F, Stazi MA, Pino A, Visconti A (2007). Serum chemical elements and oxidative status in Alzheimer’s disease, Parkinson disease and multiple sclerosis. Neurotoxicology.

[5] Fraga CG (2005). Relevance, essentiality and toxicity of trace elements in human health. Mol Asp Med.

[6] Shohag H, Ullah A, Qusar S, Rahman M, Hasnat A (2012). Alterations of serum zinc, copper, manganese, iron, calcium, and magnesium concentrations and the complexity of interelement relations in patients with obsessive-compulsive disorder. Biol Trace Elem Res.

[7] Mustak MS, Rao TS, Shanmugavelu P, Sundar NM, Menon RB, Rao RV (2008). Assessment of serum macro and trace element homeostasis and the complexity of inter-element relations in bipolar mood disorders. Clin Chim Acta.

[8] Nahar Z, Azad MA, Rahman MA, Rahman MA, Bari W, Islam SN (2010). Comparative analysis of serum manganese, zinc, calcium, copper and magnesium level in panic disorder patients. Biol Trace Elem Res.

[9] Fukushima T, Tan X, Luo Y, Kanda H (2010). Relationship between blood levels of heavy metals and Parkinson's disease in China. Neuroepidemiology.

[10] Błażewicz A, Liao KY, Liao HH (2017). Alterations of hair and nail content of selected trace elements in nonoccupationally exposed patients with chronic depression from different geographical regions. Biomed Res Int.

[11] Jung KI, Ock SM, Chung JH (2010). Associations of serum Ca and Mg levels with mental health in adult women without psychiatric disorders. Biol Trace Elem Res.

[12] Szkup M, Jurczak A, Brodowska A (2017). Analysis of relations between the level of mg, Zn, ca, cu, and Fe and depressiveness in postmenopausal women. Biol Trace Elem Res.

[13] Tarleton EK, Littenberg B, MacLean CD, Kennedy AG, Daley C (2017). Role of magnesium supplementation in the treatment of depression: a randomized clinical trial. PLoS One.

[14] da Silva F, Copper WR, Silva F, Williams R (1999). Extracytoplasmic oxidases and matrix formation. The biological chemistry of the elements. The inorganic chemistry of life.

[15] Frewin R, Henson A, Provan D (1997). ABC of clinical haematology. Iron deficiency anaemia. BMJ.

[16] Piao YS, Lian TH, Hu Y (2017). Restless legs syndrome in Parkinson disease: clinical characteristics, abnormal iron metabolism and altered neurotransmitters. Sci Rep.

[17] Jellen LC, Beard JL, Jones BC (2009). Systems genetics analysis of iron regulation in the brain. Biochimie.

[18] Chen P, Chakraborty S, Mukhopadhyay S, Lee E, Paoliello MM, Bowman AB, Aschner M (2015). Manganese homeostasis in the nervous system. J Neurochem.

[19] Takeda A (2003). Manganese action in brain function. Brain Res Brain Res Rev.

[20] Li SJ, Jiang L, Fu X, Huang S, Huang YN, Li XR, Chen JW, Li Y, Luo HL, Wang F, Ou SY, Jiang YM (2014). Pallidal index as biomarker of manganese brain accumulation and associated with manganese levels in blood: a meta-analysis. PLoS One.

[21] Grünec ker B, Kaltwasser SF, Zappe AC, Bedenk BT, Bicker Y, Spoormaker VI, Wotjak CT, Czisch M (2013). Regional specificity of manganese accumulation and clearance in the mouse brain: implications for manganese-enhanced MRI. NMR Biomed.

[22] Rotruck JT, Pope AL, Ganther HE, Swanson AB, Hafeman DG, Hoekstra WG (1973). Selenium: biochemical role as a component of glutathione peroxidase. Science.

[23] Plum LM, Rink L, Haase H (2010). The essential toxin: impact of zinc on human health. Int J Environ Res Public Health.

[24] Nowak G (2001). Does interaction between zinc and glutamate system play a significant role in the mechanism of antidepressant action?. Acta Pol Pharm.

[25] Prashanth L, Kattapagari KK, Chitturi RT, Baddam VR, Prasad LK (2015). A review on role of essential trace elements in health and disease. J NTR Univ Health Sci.

[26] Livesay DR, Jambeck P, Rojnuckarin A, Subramaniam S (2003). Conservation of electrostatic properties within enzyme families and superfamilies. Biochemistry.

[27] Cao X, Antonyuk SV, Seetharaman SV, Whitson LJ, Taylor AB, Holloway SP, Strange RW, Doucette PA, Valentine JS, Tiwari A, Hayward LJ, Padua S, Cohlberg JA, Hasnain SS, Hart PJ (2008). Structures of the G85R variant of SOD1 in familial amyotrophic lateral sclerosis. J Biol Chem.

[28] Stern BR, Solioz M, Krewski D, Aggett P, Aw TC, Baker S, Crump K, Dourson M, Haber L, Hertzberg R, Keen C, Meek B, Rudenko L, Schoeny R, Slob W, Starr T (2007). Copper and human health: biochemistry, genetics, and strategies for modeling dose-response relationships. J Toxicol Environ Health B Crit Rev.

[29] Amin MN, Liza KF, Sarwar MS, Ahmed J, Adnan MT, Chowdhury MI, Hossain MZ, Islam MS (2015). Effect of lipid peroxidation, antioxidants, macro minerals and trace elements on eczema. Arch Dermatol Res.

[30] Sarwar MS, Ahmed S, Ullah MS, Kabir H, Rahman GK, Hasnat A, Islam MS (2013). Comparative study of serum zinc, copper, manganese, and iron in preeclamptic pregnant women. Biol Trace Elem Res.

[31] Anderson JG, Erikson KM, Preedy V, Watson R, Martin C (2011). The importance of trace elements for neurological function. Handbook of behavior, food and nutrition.

[32] Wacker WE, Parisi AF (1968). Magnesium metabolism. N Engl J Med.

[33] Eby GA, Eby KL (2006). Rapid recovery from major depression using magnesium treatment. Med Hypotheses.

[34] Frizel D, Coppen A, Marks V (1969). Plasma magnesium and calcium in depression. Br J Psychiatry.

[35] Zieba A, Kata R, Dudek D, Schlegel-Zawadzka M, Nowak G (2000). Serum trace elements in animal models and human depression: part III. Magnesium. Relationship with copper. Hum Psychopharmacol.

[36] Derom ML, Sayón-Orea C, Martínez-Ortega JM, Martínez-González MA (2013). Magnesium and depression: a systematic review. Nutr Neurosci.

[37] Rajizadeh A, Mozaffari-Khosravi H, Yassini-Ardakani M, Dehghani A (2016). Serum magnesium status in patients subjects with depression in the City of Yazd in Iran 2013-2014. Biol Trace Elem Res.

[38] Schlegel-Zawadzka M, Zięba A, Dudek D, Krośniak M, Szymaczek M, Nowak G (1999). Serum trace elements in animal models and human depression. Part II. Cooper. Hum Psychopharmacol Clin Exp.

[39] Arredondo M, Núñez MT (2005). Iron and copper metabolism. Mol Asp Med.

[40] Pfeiffer CC, LaMola S (1983). Zinc and manganese in the schizophrenias. J Orthomol Psychiatry.

[41] Benton D (2002). Selenium intake, mood and other aspects of psychological functioning. Nutr Neurosci.

[42] Maes M, Galecki P, Chang YS, Berk M (2011). A review on the oxidative and nitrosative stress (O&NS) pathways in major depression and their possible contribution to the neurodegenerative processes in the illness. Prog Neuro-Psychopharmacol Biol Psychiatry.

[43] Grønli O, Kvamme JM, Friborg O, Wynn R (2013). Zinc deficiency is common in several psychiatric disorders. PLoS One.

[44] Mustak MS, Rao TS, Shanmugavelu P, Sundar NM, Menon RB, Rao RV, Rao KS (2008). Assessment of serum macro and trace element homeostasis and the complexity of inter-element relations in bipolar mood disorders. Clin Chim Acta.

[45] Siwek M, Szewczyk B, Dudek D, Styczeń K, Sowa-Kućma M, Młyniec K, Siwek A, Witkowski L, Pochwat B, Nowak G (2013). Zinc as a marker of affective disorders. Pharmacol Rep.

[46] Lam MH, Chau SW, Wing YK (2009). High prevalence of hypokalemia in acute psychiatric inpatients. Gen Hosp Psychiatry.

[47] Schaafsma G (1997). Bioavailability of calcium and magnesium. Eur J Clin Nutr.

[48] Gibson RS (2007). The role of diet- and host-related factors in nutrient bioavailability and thus in nutrient-based dietary requirement estimates. Food Nutr Bull.

[49] Freeland-Graves JH, Sanjeevi N, Lee JJ (2015). Global perspectives on trace element requirements. J Trace Elem Med Biol.

[50] de Romaña DL, Olivares M, Uauy R, Araya M (2011). Risks and benefits of copper in light of new insights of copper homeostasis. J Trace Elem Med Biol.

[51] Schlegel-Zawadzka M, Nowak G (2000). Alterations in serum and brain trace element levels after antidepressant treatment. Part II. Copper. Biol Trace Elem Res.

[52] Nowak G, Schlegel-Zawadzka M (1999). Alterations in serum and brain trace element levels after antidepressant treatment: part I. Zinc. Biol Trace Elem Res.

[53] Christensen JM, Lead KJ, Seiler HG, Siegel A, Sigel H (1994). Handbook on metals in clinical and analytical chemistry.

[54] Caroli S, Alimonti A, Coni E, Petrucci F, Senofonte O, Violante N (1994). The assessment of reference values for elements in human biological tissues and fluids: a systematic review. Crit Rev Anal Chem.

